# Cortical activation patterns mediating motor recovery: a randomized controlled fNIRS study of rTMS-integrated dual-task training in stroke survivors

**DOI:** 10.3389/fneur.2026.1785709

**Published:** 2026-04-10

**Authors:** Yilan Sheng, Jia Han

**Affiliations:** 1 Department of Rehabilitation, Shanghai University of Sport, Shanghai, China; 2 Department of Rehabilitation, Shanghai General Hospital, Shanghai Jiaotong University, Shanghai, China

**Keywords:** dual-task training, functional near-infrared spectroscopy (fNIRS), gait recovery, repetitive transcranial magnetic stimulation (rTMS), stroke, supplementary motor area

## Abstract

**Background:**

Stroke remains a leading cause of long-term disability worldwide, imposing a substantial burden on individuals and healthcare systems. The recovery of gait and balance is often hindered by cognitive-motor interference, necessitating effective rehabilitation strategies.

**Objectives:**

We aimed to evaluate the synergistic efficacy of repetitive transcranial magnetic stimulation (rTMS) combined with cognitive-motor dual-task training on lower limb motor recovery in stroke survivors.

**Methods:**

A total of 150 stroke survivors were randomized into three groups: the single-task walking group (Group 1, *n* = 48), the cognitive-motor dual-task walking group (Group 2, *n* = 52), and the rTMS-integrated dual-task group (Group 3, *n* = 50). Clinical outcomes and biomechanical parameters were assessed across four timepoints (baseline, 1, 3, and 6 months post-intervention). Analysis of covariance (ANCOVA) was utilized to control for lesion characteristics. Functional near-infrared spectroscopy (fNIRS) was employed to monitor hemodynamic responses in the supplementary motor area (SMA) and premotor cortex (PMC) during dual-task walking.

**Results:**

A two-way repeated measures ANOVA revealed a highly robust group × time interaction (*p* < 0.001). At the 6-month follow-up, Group 3 demonstrated significantly superior recovery compared to Groups 1 and 2. Specifically, Group 3 achieved the highest walking speed (0.94 ± 0.15 m/s) with an extremely large effect size (Cohen’s *d* = 2.07), and the greatest absolute improvement in step length (19.0 cm, *p* < 0.001). The clinical responder rates based on the minimum clinically important difference (MCID) in Group 3 were exceptional: 94.0% for gait speed and 96.0% for step length, both significantly higher than in the control groups. fNIRS imaging revealed that Group 3 exhibited significantly higher oxyhemoglobin (HbO) concentrations in the SMA (*p* = 0.010) and PMC (*p* = 0.008) compared to Group 1. Crucially, partial correlation analysis indicated that while the increase in SMA activation supported motor recovery, this relationship was deeply coupled with the increase in gait velocity, shifting to a marginal trend when controlling for speed changes (*r* = 0.255, *p* = 0.077).

**Conclusion:**

The integration of rTMS with cognitive-motor dual-task training yields superior improvements in gait speed, stride mechanics, and balance compared to conventional therapies. This enhanced recovery is mechanistically supported by the upregulation of the supplementary motor area (SMA), suggesting that rTMS facilitates the central reorganization required for complex motor planning in concert with improved gait execution.

## Introduction

Stroke is a global disease characterized by high morbidity, mortality, and disability (1). Although approximately 70% of stroke patients survive the acute phase, survivors frequently contend with persistent sequelae, including motor deficits, cognitive impairment, and sensory dysfunction (2). Among these, motor impairment, particularly gait and balance dysfunction, is a common and debilitating complication that significantly compromises activities of daily living (ADL) and overall quality of life. The restoration of motor function requires a multifaceted, multidisciplinary approach and remains a formidable clinical challenge (3).

In daily life, the execution of functional activities necessitates the seamless coordination of motor tasks and cognitive processes. This “dual-task” requirement is essential for maintaining postural control during dynamic actions such as walking or navigating obstacles (4). Consequently, stroke survivors often encounter significant challenges when performing simultaneous tasks, as the competition for shared neural resources leads to cognitive-motor interference (5). Balance is the fundamental prerequisite for attaining an upright posture, encompassing sitting, standing, and gait, and is indispensable for the safe performance of ADL. Improving balance function is critical not only for functional independence but also for mitigating the high risk of falls in this population. Emerging evidence suggests that task-specific rehabilitation can effectively enhance balance and dual-task performance during stroke recovery (6–9).

Guided by the principles of neuroplasticity, contemporary stroke rehabilitation aims to activate the damaged sensorimotor cortex, promote cortical remodeling, and facilitate the reconstruction of neural pathways to restore interhemispheric balance (10). Repetitive transcranial magnetic stimulation (rTMS) has emerged as a prominent non-invasive brain stimulation (NIBS) technique in clinical rehabilitation. By delivering pulsed magnetic fields to specific cortical targets, rTMS induces electrical currents that alter membrane potentials and modulate neural activity (11). Through this neuromodulatory effect, rTMS can regulate the excitability of both the ipsilesional and contralesional hemispheres, guiding functional reorganization and promoting neurological recovery (12). Beyond its established role in increasing motor neuron output and muscular force production (13), rTMS has also demonstrated potential in enhancing cognitive domains such as language, memory, and executive function (14).

Despite the potential of rTMS and dual-task training individually, the synergistic effects of combining these interventions remain underexplored. In this study, we investigated the efficacy of a combined protocol involving rTMS and cognitive-motor dual-task walking training on the recovery of lower limb motor function and gait stability. Furthermore, we employed functional near-infrared spectroscopy (fNIRS) to explore the neurophysiological mechanisms of cortical activation. Previous neuroimaging studies have established that while healthy individuals rely on highly automated, subcortical networks for routine steady-state walking, stroke survivors exhibit a compensatory reliance on heightened cortical activation, particularly within the supplementary motor area (SMA), premotor cortex (PMC), and prefrontal cortex, to consciously control gait and manage cognitive-motor interference (15–17). Building upon this established paradigm, our study specifically focuses on how these interventions drive the neural reorganization required for the transition from single-task to complex dual-task walking.

## Methods

This study was approved by the Ethics Committee of the Shanghai University of Sport and the Ningbo Ninth Hospital (Jiangbei Branch of Ningbo First Hospital) (Approval No. 2022LIW08). The protocol was conducted in accordance with the Declaration of Helsinki and registered in the Chinese Clinical Trial Registry. Methodological reporting followed the EQUATOR Network guidelines, specifically the Consensus on Exercise Reporting Template (CERT). All participants provided written informed consent prior to enrollment (18).

### Participants

Stroke participants were recruited from the Ledu Rehabilitation Center of Shanghai First People’s Hospital, Shanghai Yangzhi Rehabilitation Hospital, and Ningbo Ninth Hospital between June 2023 and December 2023. Inclusion criteria: (1) Clinical diagnosis of first-ever or recurrent stroke confirmed by CT/MRI (19); (2) Age over 50 years (20); (3) Post-stroke duration of 6–24 months; (4) Functional Independence Measure (FIM) walking score ≥ 5 [assessed without physical human assistance, though habitual use of a cane or ankle-foot orthosis was permitted if used consistently across all assessments] (21); (5) Willingness to comply with the treatment protocol. Exclusion criteria: (1) Progressive or unstable stroke; (2) Severe uncontrolled systemic diseases (e.g., uremia, CHD); (3) Cognitive impairment (MMSE score <27, adjusted for education: university ≤24, secondary ≤22, primary ≤20, illiteracy ≤17 (22)); (4) Significant hearing or language deficits preventing cooperation.

### Estimation of clinical trial sample size

The sample size was estimated using G Power 3.1.9.7. For a one-way ANOVA with an effect size of 0.25, power of 0.80, and α = 0.05, a minimum of 159 cases was initially targeted. Initially, a total of 176 participants were recruited. In practice, 150 participants completed the full intervention and assessment cycle (representing an approximate 15% attrition rate, primarily due to transportation difficulties or unrelated medical issues). Participants were randomly assigned to three groups using block randomization (block size = 6, seed = 2023) via Stata 11.0, stratified by age and baseline motor severity. Allocation was performed in a 1:1:1 ratio: Group 1 (single-task, *n* = 48); Group 2 (dual-task, *n* = 52); Group 3 (rTMS, *n* = 50). The study employed an assessor-blinded design. While therapists and patients could not be fully blinded, outcome assessors and data analysts remained blinded to group allocation.

### Interventions

All groups received 4 weeks of standard rehabilitation (5 days/week), which was administered as an intensive day-hospital program suitable for chronic stroke survivors (6–24 months post-stroke) to maximize recovery plateau breakthroughs. This standard care comprised 60 min of conventional physical and occupational therapy per session. In addition to standard care, specific experimental interventions (30 min per session) were applied according to group allocation. Participants in Group 1 (single-task) performed gait training using the Das Zebris Rehawalk^®^ System (23) focusing solely on stride correction without cognitive interference. Conversely, Group 2 (dual-task) engaged in cognitive-motor dual-task walking, which combined motor tasks such as obstacle navigation with simultaneous cognitive challenges, including Verbal Fluency Tests (VFT) (24) and Stroop interference tasks (25). Finally, participants in Group 3 (rTMS) received high-frequency rTMS (3 Hz, 90% MT, 1000 pulses/session) using the Magneuro 60/100 system immediately prior to engaging in the identical dual-task walking protocol described for Group 2 (26). For the rTMS application, the magnetic coil was positioned over the Cz area (based on the international 10–20 EEG system) to target the bilateral lower limb motor representation. To ensure optimal and safe stimulation intensity throughout the intervention, the resting motor threshold (RMT) was initially determined using motor evoked potentials (MEPs) monitored from the affected tibialis anterior muscle, and this threshold was rigorously re-assessed on a weekly basis.

### Outcome measures

To ensure a rigorous and multi-dimensional evaluation of the intervention’s efficacy, all participants underwent comprehensive clinical and biomechanical assessments at four distinct timepoints: baseline, 1 month, 3 months, and 6 months post-intervention. Clinical gait and motor function were quantified using the 10-Meter Walk Test (10MWT) (27) and the lower extremity portion of the Fugl-Meyer Assessment (FMA-LE). The 10MWT was conducted by measuring the time required for participants to traverse a marked 10-meter path at their maximum safe walking speed. Functional mobility and cognitive-motor integration were scrutinized using the Timed Up and Go Test (TUGT) (27) and its dual-task variant (DTUGT, employing serial subtractions of 7). Furthermore, static and dynamic balance capabilities were quantified using the Sensory Organization Test (SOT) and Composite Equilibrium Score (CES) via dynamic posturography, supplemented by the Falls Efficacy Scale-International (FES-I).

A comprehensive biomechanical profile of gait quality was established using the Zebris Rehawalk^®^ system. For safety, participants wore an overhead body-weight support harness with zero unweighting and were instructed not to hold the handrails unless they experienced a loss of balance. The system extracted advanced parameters, including step length, step speed, stride height, symmetry index, and touch time (defined as the duration of the foot’s contact with the ground during the stance phase).

### Functional near-infrared spectroscopy acquisition

Cerebral hemodynamic responses, specifically changes in oxy-hemoglobin (HbO) concentrations, were captured during dual-task walking using the NIRSport2 wireless portable system (NIRX, United States). The monitoring focused on predefined regions of interest (ROIs) critical for motor planning and execution, specifically the supplementary motor area (SMA) and the premotor cortex (PMC). To achieve a robust signal-to-noise ratio, the fNIRS procedure used a block-design protocol lasting approximately 10 min, in which dual-task walking was repeated across four alternating blocks (30 s task, 30 s rest). Cortical activation levels were quantified using beta values from the general linear model (GLM).

### Statistical analysis

All statistical analyses were executed using SPSS version 20.0 (IBM Corp., Armonk, NY) and R version 4.2.1. Quantitative data are presented as mean ± standard deviation (SD). Initial group comparability was assessed using one-way ANOVA and chi-square tests. To rigorously address potential anatomical confounding effects (as queried during peer review), analysis of covariance (ANCOVA) was employed to evaluate whether the lesioned hemisphere (left vs. right) or specific lesion locations had a significant main or interaction effect on the primary outcomes.

Longitudinal efficacy was analyzed using a two-way repeated measures ANOVA to evaluate the main effects of group, time (baseline, 1 m, 3 m, 6 m), and their group × time interaction. Tukey’s HSD *post-hoc* tests were performed for pairwise comparisons. Standardized effect sizes (Cohen’s *d*) with 95% confidence intervals were calculated using the effectsize package in R to quantify the magnitude of treatment differences.

The neural drivers of clinical recovery were explored through correlation analysis. Critically, to determine if SMA activation changes were an independent neural driver or merely secondary to increased gait velocity, a partial correlation analysis was conducted, controlling for the change in 10MWT speed ( Δ 10MWT). Finally, clinical relevance was evaluated via responder analyses based on the established minimum clinically important differences (MCID). Participants were categorized as responders if they achieved an absolute improvement of ≥ 5 cm for step length or ≥ 0.10 m/s for gait speed. Statistical significance for all tests was set at *p* < 0.05.

## Results

### Baseline demographic and clinical profile

A total of 150 stroke survivors completed the intervention and follow-up assessments, distributed across the single-task (Group 1, *n* = 48), dual-task (Group 2, *n* = 52), and rTMS-integrated (Group 3, *n* = 50) cohorts. As detailed in Table 1, the three groups demonstrated high comparability across all demographic and clinical domains at study entry. The mean age was 61.5 ± 9.1 years for Group 1, 62.5 ± 7.4 for Group 2, and 62.6 ± 5.8 for Group 3 (*p* = 0.727). Baseline assessments of stroke duration, NIHSS, MMSE scores, the number of previous stroke incidents, exact lesion anatomical locations, and the affected hemisphere showed no significant differences (all *p* > 0.05). Analysis of covariance (ANCOVA) was employed to adjust for minor initial functional variances, and specifically revealed that the lesioned hemisphere (left vs. right) had no significant main effect or interaction with the treatment group on the recovery outcomes (*p* = 0.999), ensuring a statistically uniform starting point for efficacy evaluation across different brain injury profiles.

**Table 1:** Baseline demographic, clinical, and lesion characteristics of the study participants.

Variable	Single-task (*n* = 48)	Dual-task (*n* = 52)	rTMS-integrated (*n* = 50)	*p*-value
Age (years, mean ± SD)	61.5 ± 9.1	62.5 ± 7.4	62.6 ± 5.8	**0.723**
Sex (male, *n*, %)	27 (56.2%)	32 (61.5%)	32 (64.0%)	**0.726**
Lesion hemisphere (left, *n*, %)	28 (58.3%)	26 (50.0%)	31 (62.0%)	**0.455**
Lesion location (basal ganglia, *n*, %)	21 (43.8%)	21 (40.4%)	19 (38.0%)	**0.833**
Recurrent stroke (≥ incidents, *n*, %)	25 (52.1%)	26 (50.0%)	22 (44.0%)	**0.706**
Stroke duration (months, mean ± SD)	11.9 ± 4.1	10.7 ± 3.8	11.5 ± 3.4	**0.251**
NIHSS score (mean ± SD)	9.9 ± 1.6	9.8 ± 2.0	10.3 ± 1.9	**0.305**
MMSE score (mean ± SD)	26.8 ± 1.5	27.2 ± 1.3	27.4 ± 1.4	**0.141**
FMA-LE score	24.5 ± 3.2	25.1 ± 2.9	24.8 ± 3.1	0.614

Data are presented as mean ± standard deviation (SD) or number (percentage). *p*-values indicate inter-group differences derived from one-way ANOVA for continuous variables or chi-square tests for categorical variables. Variables include the number of previous stroke incidents, lesioned hemisphere (left/right), and specific lesion anatomical locations. Baseline functional scores (10MWT, FMA-LE) were subsequently adjusted using ANCOVA to control for initial variances. BMI, body mass index; SBP, systolic blood pressure; DBP, diastolic blood pressure; NIHSS, National Institutes of Health Stroke Scale; MMSE, Mini-Mental State Examination. Bold values indicate statistically significant differences (*p* < 0.05).

### Walking function and step length gains

**Figure 1:**
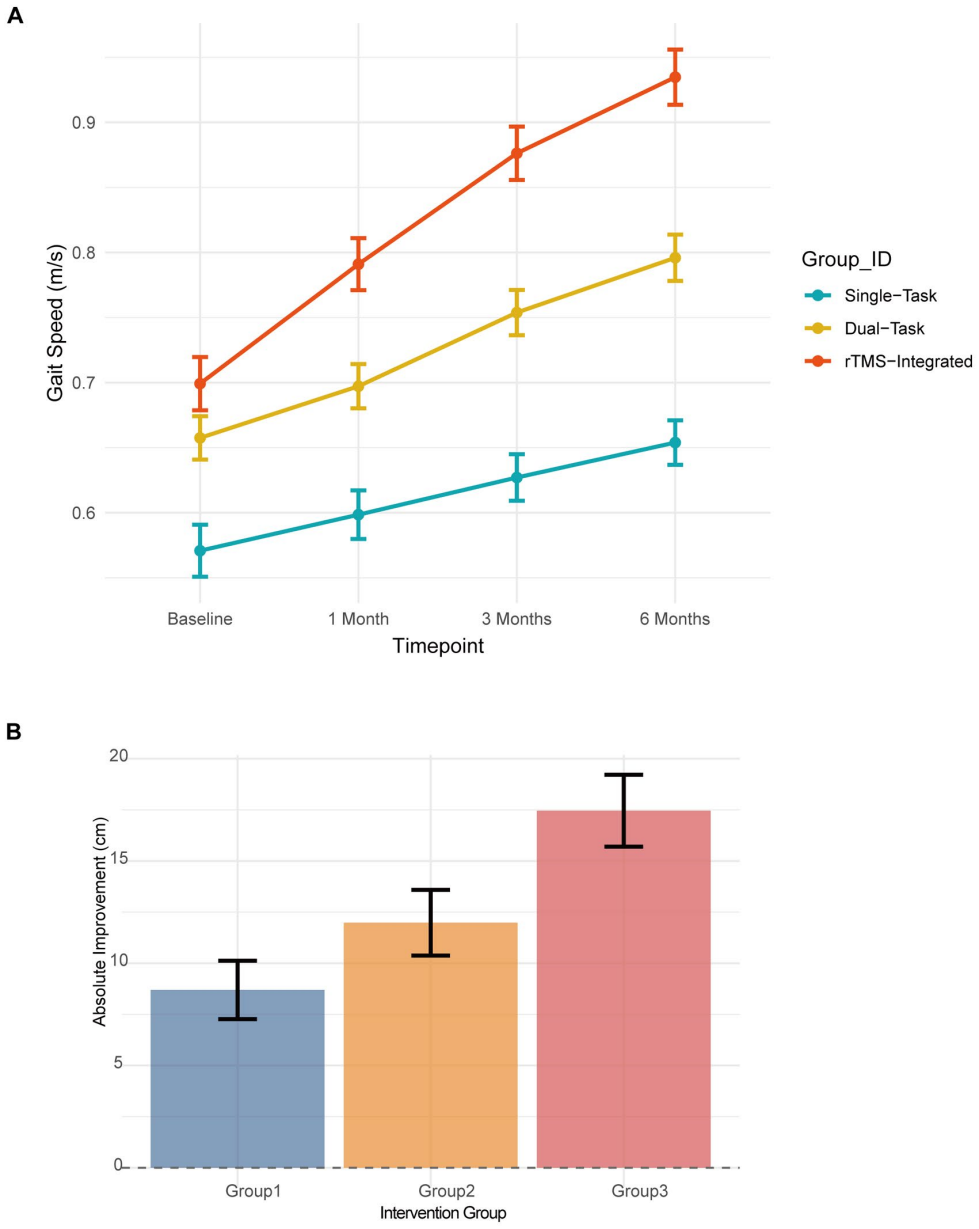
Recovery of walking function. **(A)** Longitudinal trajectories of walking speed (10MWT) across four evaluation timepoints (baseline, 1, 3, and 6 months). Group 3 exhibits the steepest recovery slope (*p* < 0.001 for group × time interaction). **(B)** Absolute improvement in step length at 6 months. Error bars represent 95% CIs.

Analysis of primary gait outcomes via a two-way repeated measures ANOVA tracking four distinct timepoints (baseline, 1 month, 3 months, and 6 months) revealed a highly robust group × time interaction (*p* < 0.001), indicating that the recovery trajectories were significantly influenced by the type of intervention received. As illustrated in Figure 1A, the 10-Meter Walk Test (10MWT) results showed that Group 3 displayed a markedly steeper recovery slope compared to the control groups, reaching 0.94 ± 0.15 m/s at 6 months (Table 2). Furthermore, the absolute improvement in step length for Group 3 reached 19.0 ± 5.0 cm, representing a 49.1% relative gain from baseline (Figure 1B). This improvement was significantly greater than the gains observed in Group 2 (13.9 ± 3.5 cm) and Group 1 (9.1 ± 4.3 cm), with *post-hoc* Tukey HSD tests confirming extreme statistical significance for the rTMS-integrated protocol (Group 3 vs. Group 1: *p* < 0.001).

**Table 2:** Summary of clinical and functional outcome measures across four assessment timepoints.

Outcome measure	Group	Baseline	1-month	3-months	6-months	Δ change	Time effect *p*	Group × time *p*
10MWT (m/s)	Group 1 (single-task)	0.58 ± 0.12	0.61 ± 0.12	0.63 ± 0.12	0.65 ± 0.12	0.08 ± 0.05	**<0.001**	**<0.001**
	Group 2 (dual-task)	0.61 ± 0.11	0.69 ± 0.12	0.75 ± 0.12	0.80 ± 0.13	0.14 ± 0.06		
	Group 3 (rTMS)	0.59 ± 0.13	0.73 ± 0.14	0.85 ± 0.14	0.94 ± 0.15	0.24 ± 0.08		
Step length (cm)	Group 1 (single-task)	38.5 ± 6.2	42.1 ± 6.1	45.3 ± 6.0	47.6 ± 5.9	9.1 ± 4.3	**<0.001**	**< 0.001**
	Group 2 (dual-task)	39.1 ± 5.8	44.7 ± 5.9	49.5 ± 6.0	53.0 ± 6.1	13.9 ± 3.5		
	Group 3 (rTMS)	38.8 ± 6.0	46.4 ± 6.2	53.0 ± 6.4	57.8 ± 6.5	19.0 ± 5.0		
FMA-LE (0–34)	Group 1 (single-task)	24.5 ± 3.2	26.1 ± 3.3	27.4 ± 3.4	28.4 ± 3.5	3.9 ± 2.8	**<0.001**	**< 0.001**
	Group 2 (dual-task)	25.1 ± 2.9	27.3 ± 3.0	29.1 ± 3.1	30.5 ± 3.2	5.4 ± 2.7		
	Group 3 (rTMS)	24.8 ± 3.1	28.7 ± 3.0	32.1 ± 2.9	34.5 ± 2.8	9.7 ± 1.3		
TUGT (s)	Group 1 (single-task)	28.5 ± 4.5	26.8 ± 4.2	25.3 ± 4.0	24.2 ± 3.8	−4.3 ± 4.2	**<0.001**	**<0.001**
	Group 2 (dual-task)	27.8 ± 3.9	25.3 ± 3.6	23.1 ± 3.4	21.5 ± 3.2	−6.3 ± 3.5		
	Group 3 (rTMS)	28.1 ± 4.2	24.2 ± 3.7	20.8 ± 3.2	18.4 ± 2.9	−9.7 ± 3.5		
DTUGT (s)	Group 1 (single-task)	33.5 ± 5.2	32.6 ± 5.1	31.8 ± 5.0	31.2 ± 4.9	−2.3 ± 2.1	**<0.001**	**<0.001**
	Group 2 (dual-task)	32.8 ± 4.8	29.6 ± 4.5	26.9 ± 4.3	24.9 ± 4.1	−7.9 ± 3.5		
	Group 3 (rTMS)	32.9 ± 5.4	28.1 ± 4.9	24.0 ± 4.5	21.0 ± 4.2	−11.9 ± 3.8		

Values are expressed as mean ± SD. The table details the longitudinal changes in the 10-Meter Walk Test (10MWT), Fugl-Meyer Assessment-Lower Extremity (FMA-LE), Timed Up and Go Test (TUGT), and dual-task TUGT (DTUGT) across the three groups at baseline, 1 month, 3 months, and 6 months post-intervention. Δ represents the absolute change from baseline to 6 months. Significance levels refer to the group × time interaction effect from the two-way repeated measures ANOVA. rTMS, repetitive transcranial magnetic stimulation. Bold values indicate statistically significant differences (*p* < 0.05).

### Recovery of lower extremity motor function

**Figure 2:**
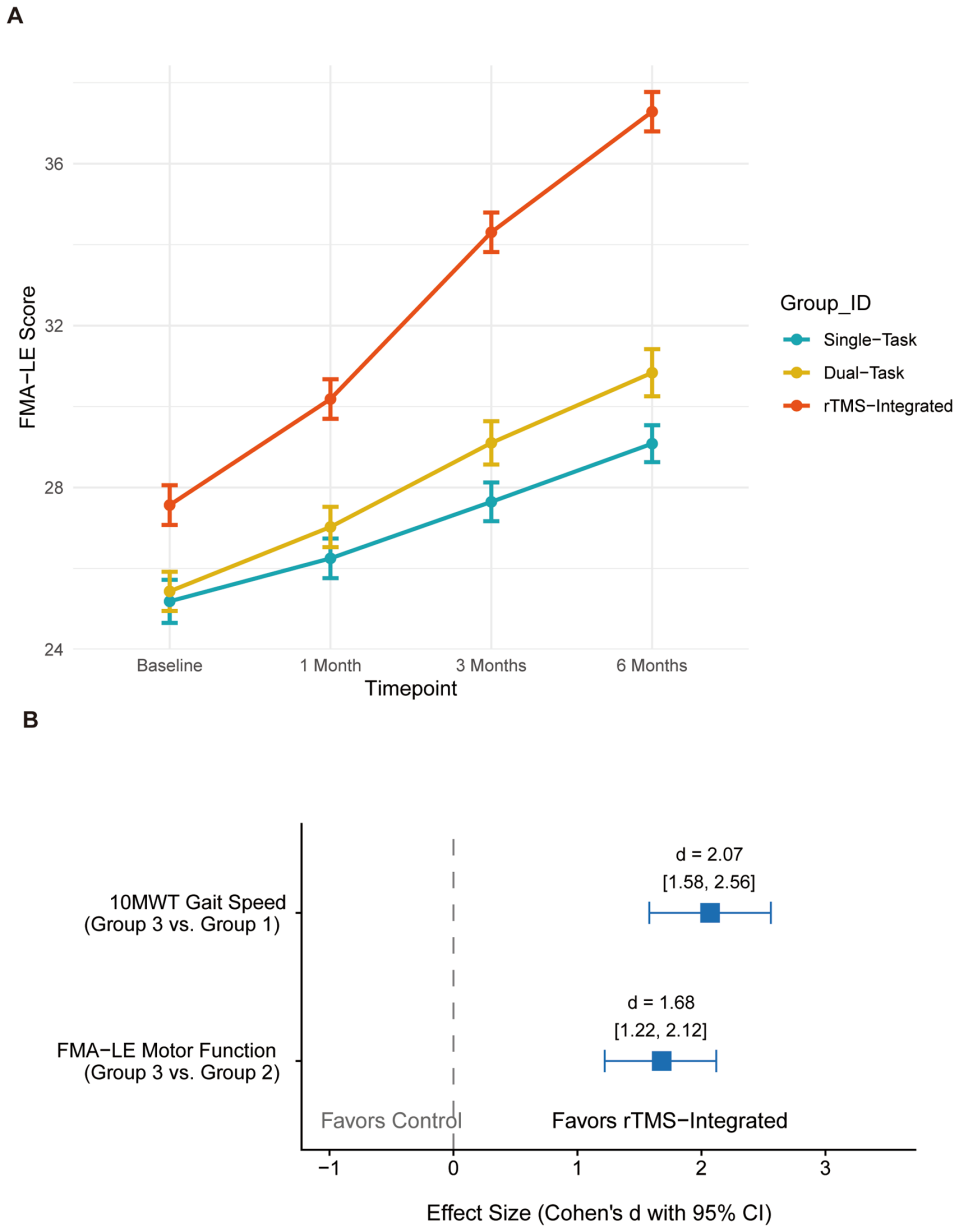
Lower extremity motor outcomes and treatment efficacy. **(A)** Time course of Fugl-Meyer Assessment-Lower Extremity (FMA-LE) scores across the four assessment timepoints. **(B)** Forest plot of effect size estimates (Cohen’s *d*) with 95% CIs for gait speed and motor function at 6 months. Positive values favor the rTMS intervention over control groups (e.g., *d* = 2.07 vs. Group 1 for 10MWT).

The recovery of motor synergism in the hemiplegic limb, assessed via the Fugl-Meyer Assessment-Lower Extremity (FMA-LE) across the four timepoints, demonstrated a significant interaction effect (*p* < 0.001), which is visualized in the longitudinal trajectory plots in Figure 2A. Group 3 participants achieved a mean functional gain of 9.7 ± 1.3 points at the 6-month mark, constituting a 36.0% improvement relative to baseline. The effect size estimates, visualized in the forest plot (Figure 2B), confirmed a substantial, favorable outcome for the rTMS intervention over controls. Specifically, calculations yielded extremely large effect sizes for the combined intervention, with a Cohen’s *d* of 2.07 for gait speed (Group 3 vs. Group 1) and 1.68 for motor function (Group 3 vs. Group 2), with 95% CIs remaining entirely on the positive side of the efficacy threshold.

### Functional mobility, dual-task interference, and clinical responders

**Figure 3:**
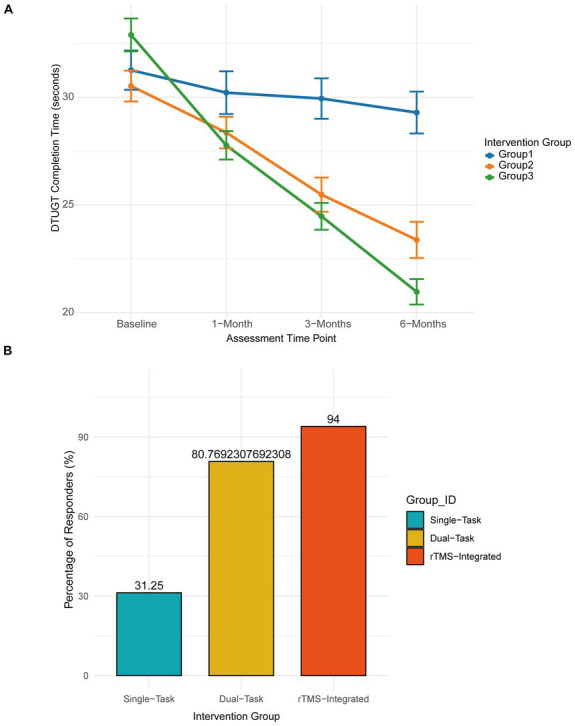
Dual-task mobility and clinical responder analysis. **(A)** Reduction in dual-task TUGT (DTUGT) completion time over 6 months. **(B)** Clinical responder analysis demonstrating the percentage of participants achieving the minimum clinically important difference (MCID) for gait speed ( ≥ 0.10 m/s) at 6 months, highlighting a 94.0% response rate in Group 3.

The assessment of functional mobility revealed that Group 3 achieved superior recovery in movement control. Time required for the standard Timed Up and Go Test significantly decreased in Group 3, falling from a baseline of approximately 28.1 ± 4.2 s to 18.4 ± 2.9 s at 6 months, indicating a robust recovery in basic mobility. While the standard Timed Up and Go Test (TUGT) times decreased significantly in Group 3, the most remarkable divergence was observed during the dual-task TUGT (DTUGT) (Figure 3A). Under the cognitive-loading condition of serial 7 s, Group 3 demonstrated a dramatic 36.2% improvement in completion time, reducing from a baseline of 32.9 ± 5.4 s to 21.0 ± 4.2 s at the 6-month follow-up. In contrast, Group 1 and Group 2 achieved only 6.6 and 24.1% improvements. To ascertain the real-world utility of these mobility gains, a responder analysis based on the minimal clinically important difference (MCID) for gait speed (≥0.10 m/s) was performed (Figure 3B). Group 3 achieved a 94.0% clinical response rate, significantly outperforming Group 2 (80.8%) and Group 1 (31.3%) (chi-square = 50.25, *p* < 0.001). These findings suggest that the integration of rTMS specifically targets and enhances the cortical resources required to manage complex tasks, effectively mitigating the interference between cognitive processing and motor execution.

### Comprehensive gait biomechanics profile

**Figure 4:**
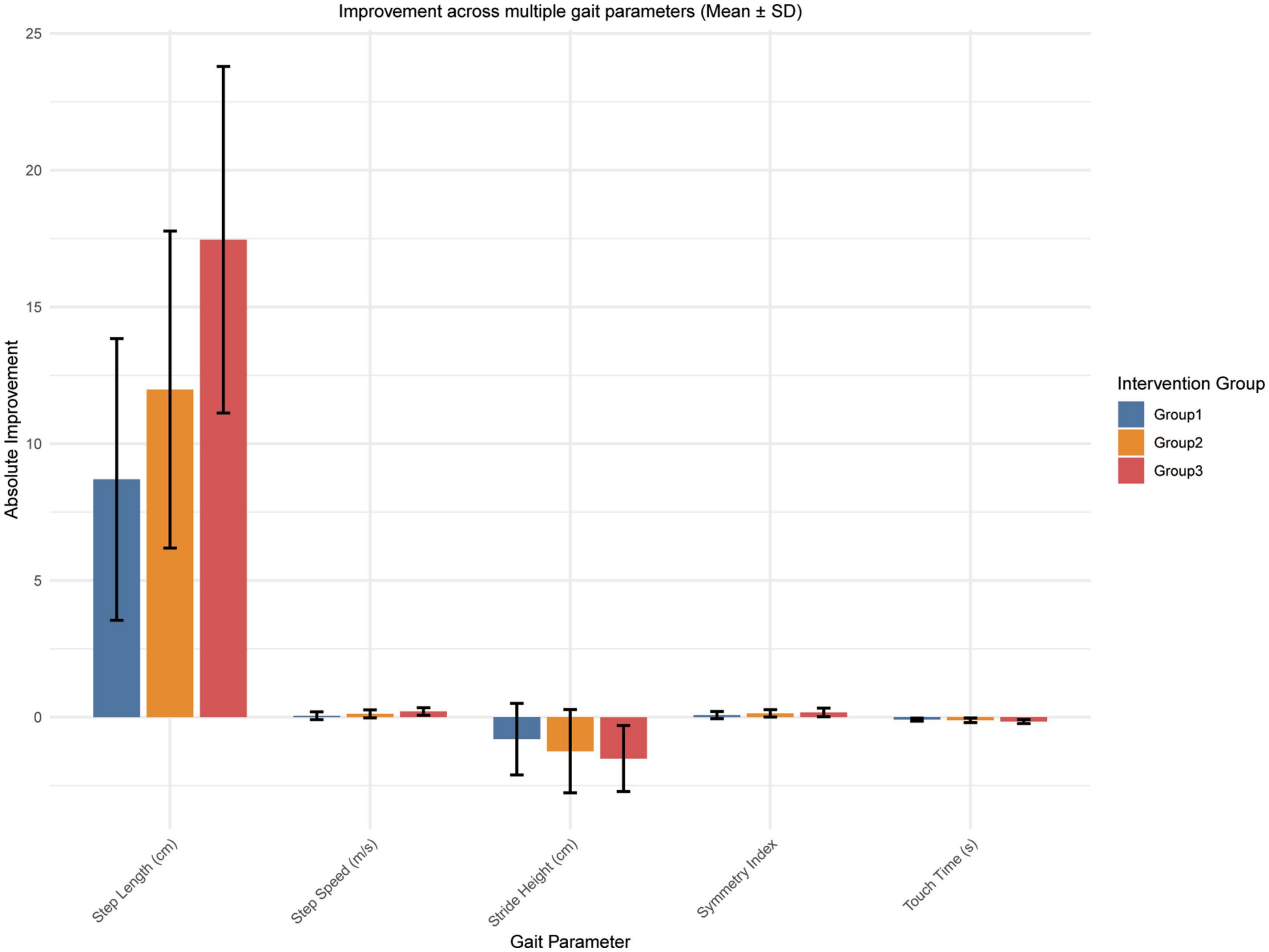
Comprehensive gait biomechanics profile. Multi-parametric analysis of gait quality improvements at 6 months. Panels display absolute changes in step length, step speed, normalized/reduced compensatory stride height, symmetry index, and touch time (stance phase stability).

Multi-parametric analysis using the Zebris system provided a profile of gait quality optimization (Figure 4). Group 3 exhibited holistic improvements across all measured parameters. Notably, this included a normalized, reduced compensatory stride height—reflecting improved and more natural foot clearance efficiency rather than pathological high-steppage and a significant reduction in touch time, suggesting enhanced stance phase stability. The symmetry index also showed significant normalization, reflecting more balanced weight-bearing between the paretic and non-paretic limbs.

### Neurophysiological mechanism: cortical activation and brain-behavior coupling

**Figure 5:**
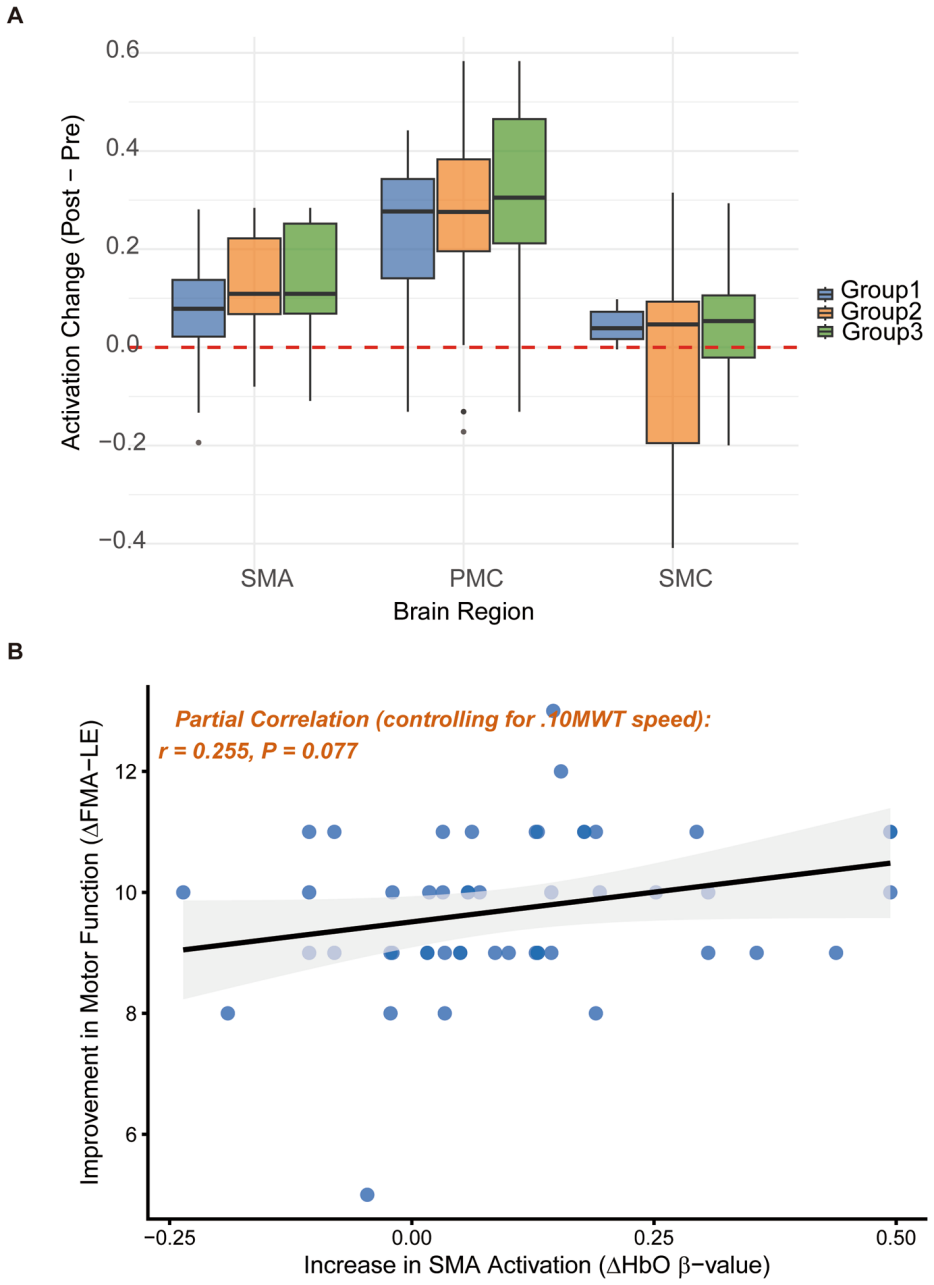
Neural mechanisms: cortical activation and brain-behavior coupling. **(A)** Box plots of hemodynamic activation changes ( Δ HbO beta values) in the SMA, PMC, and SMC during dual-task walking. **(B)** Partial correlation scatter plot between the increase in SMA activation ( Δ SMA) and clinical motor improvement, statistically controlling for the change in gait velocity ( Δ 10MWT). The relationship shows a marginal positive trend (*r* = 0.255, *p* = 0.077), indicating complex coupling between cortical planning, execution speed, and spatial motor recovery.

The functional near-infrared spectroscopy (fNIRS) data provided critical neural evidence for the observed clinical improvements, with hemodynamic responses detailed in Table 3. At 6 months, Group 3 exhibited significantly higher oxyhemoglobin (HbO) beta values in the supplementary motor area (SMA) and premotor cortex (PMC) during dual-task walking compared to the other groups (Figure 5A). The increase in SMA activation for Group 3 (=0.133 ± 0.11) was significantly greater than that of Group 1 (*p* = 0.010). Crucially, to rigorously assess the relationship between cortical reorganization and clinical outcomes, partial correlation analysis was performed (Figure 5B). When controlling for the significant increases in gait velocity ( Δ 10MWT), the positive correlation between the increase in SMA activation and clinical motor improvement remained present but shifted to a marginal trend (*r* = 0.255, *p* = 0.077). This indicates that while SMA upregulation is deeply intertwined with the capacity to walk faster, it continues to serve as a relevant neurophysiological substrate supporting the complex motor planning necessitated by the rTMS-integrated intervention.

**Table 3:** Cortical hemodynamic activation patterns (fNIRS) during dual-task walking.

Region of interest (ROI)	Metric	Group 1 (single-task) (*n* = 48)	Group 2 (dual-task) (*n* = 52)	Group 3 (rTMS) (*n* = 50)	*p*-value
SMA activation	Beta (mean)	0.076 ± 0.11	0.131 ± 0.12	**0.133 ± 0.11**	**0.01**
PMC activation	Beta (mean)	0.215 ± 0.17	0.268 ± 0.15	**0.319 ± 0.17**	**0.008**
SMC activation	Beta (mean)	0.048 ± 0.09	−0.015 ± 0.10	0.033 ± 0.08	0.152

Data represent the mean oxy-hemoglobin (HbO) beta values within the regions of interest (ROIs): supplementary motor area (SMA), premotor cortex (PMC), and sensorimotor cortex (SMC). Beta values were calculated using the general linear model (GLM). Bold values indicate statistically significant differences between Group 3 and the control groups at the 6-month assessment (*p* < 0.05). Covariate analysis confirmed no significant main or interaction effects of the lesioned hemisphere on cortical activation patterns (*p* > 0.05).

### Clinical significance and network characteristics

**Figure 6:**
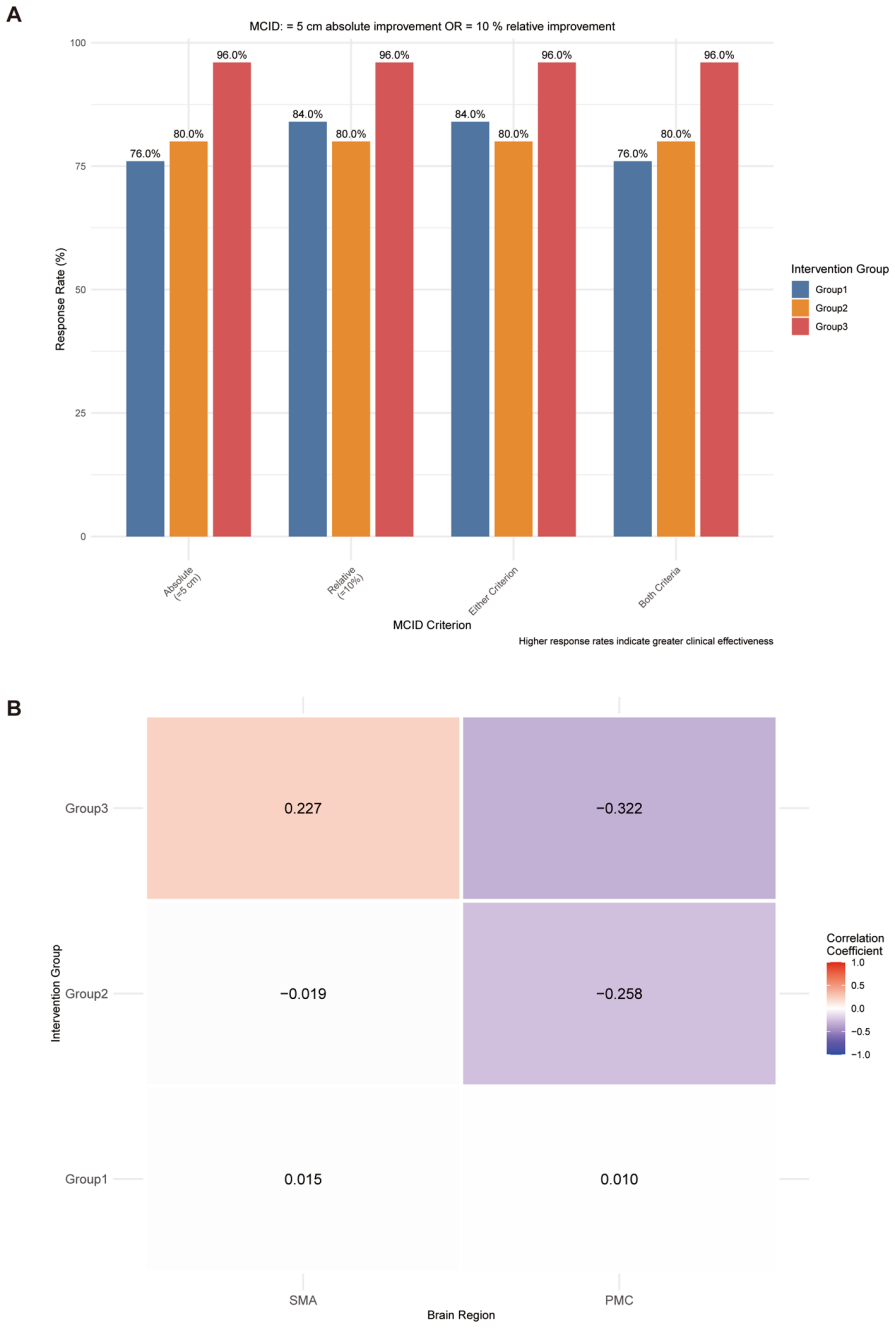
Clinical significance and network characteristics. **(A)** Clinical responder analysis based on the minimum clinically important difference (MCID) for step length, demonstrating a 96.0% response rate in Group 3. **(B)** Correlation matrix heatmap of brain region activation patterns, illustrating functional connectivity profiles.

To further evaluate the clinical significance and reliability of the treatment protocol in improving spatial gait parameters, a complementary responder analysis was performed based on the MCID for step length. As demonstrated in Figure 6, Group 3 achieved an exceptional clinical response rate of 96.0% when applying either the ≥5 cm absolute improvement or the ≥10% relative improvement criterion (Figure 6A). The correlation matrix heatmap (Figure 6B) further illustrated the distinct functional connectivity profiles, confirming that the central activation mechanism supports the observed motor rehabilitation gains.

## Discussion

In this randomized controlled trial, we investigated the differential effects of single-task training, cognitive-motor dual-task (CMDT) training, and rTMS-integrated CMDT training on functional recovery in chronic stroke survivors. The primary finding is that the combined intervention (rTMS + dual-task) yielded the most robust improvements in walking speed, gait biomechanics (specifically step length and symmetry), and dual-task processing capacity. Crucially, using fNIRS technology, we provided neurophysiological evidence that this clinical recovery is underpinned by specific cortical reorganization, particularly the upregulation of the supplementary motor area (SMA) and its intricate relationship with gait velocity.

### Synergistic effects of rTMS and dual-task training

While all groups showed improvement, the rTMS-integrated group (Group 3) significantly outperformed the dual-task (Group 2) and single-task (Group 1) groups, evidenced by exceptionally large effect sizes (Cohen’s *d* > 1.6) and a 94.0% clinical responder rate for gait speed. This superiority suggests a synergistic mechanism. Complex gait maneuvers in daily life require the simultaneous execution of motor programs and cognitive tasks, creating a “cognitive-motor interference” (CMI) (28, 29). Conventional rehabilitation often fails to address this interference. Our results confirm that while dual-task training alone (Group 2) improves this capacity better than single-task training (16), the addition of rTMS (Group 3) likely acts as a neural primer. By increasing the excitability of the motor cortex prior to training, rTMS may lower the neural threshold required for motor learning, thereby allowing patients to derive greater benefit from the subsequent high-intensity dual-task training. This aligns with the theory of “state-dependent learning,” where brain stimulation creates an optimal physiological state for neuroplasticity (12).

### Neurophysiological mechanism: the role of SMA activation

A pivotal contribution of this study is the elucidation of the central mechanism via fNIRS. An intriguing neurophysiological paradox observed in our study was that while rTMS was targeted at the central vertex (Cz) to stimulate the sensorimotor cortex (SMC), the most profound hemodynamic changes occurred in the SMA and premotor cortex (PMC), with no significant HbO alterations in the SMC itself. This phenomenon can be explained by the principles of network reorganization. Severe damage to the primary SMC often leads to a ceiling effect or localized structural deterioration, limiting its direct responsiveness (30). However, the high-frequency stimulation likely propagated through dense cortico-cortical connections to recruit higher-order motor planning regions (15, 31). Furthermore, our dual-task paradigm explicitly demands substantial cognitive-motor integration, a function primarily governed by the SMA and PMC rather than the primary motor execution areas.

Initially, we found a positive correlation between the increase in SMA activation and the improvement in step length. However, when a partial correlation analysis was conducted controlling for gait velocity ( Δ 10MWT), this relationship shifted to a marginal trend (*p* = 0.077). This nuanced finding suggests that the SMA may not be the exclusive, independent driver of spatial gait improvements; rather, its upregulation is deeply coupled with the capacity to walk faster. The SMA acts as a foundational neural substrate that facilitates the complex motor planning required to maintain higher velocities under cognitive load.

Conversely, we observed a negative correlation trend within the PMC network. The PMC is typically hyperactive in stroke survivors as a compensatory mechanism heavily reliant on external sensory cues for movement (16). The negative relationship observed at 6 months suggests that as patients in the rTMS-integrated group recovered greater automaticity and independence in walking, they successfully transitioned from relying on external PMC-driven compensation to more efficient, internal SMA-driven motor programs.

### Hemodynamic and metabolic modulation

In contrast to animal studies focusing on molecular markers (32, 33), our human study highlights the hemodynamic regulation capability of rTMS. fNIRS revealed that rTMS significantly increased local cerebral blood flow (HbO concentration) in the targeted planning regions during dual-task performance. We hypothesize that this enhanced perfusion may support the heightened metabolic demands of neuroplasticity, potentially mitigating local neural fatigue during intensive dual-task training (34, 35). This could partially explain why Group 3 could sustain performance improvements under high cognitive load (serial 7 s subtraction) where other groups faltered.

### Restoring interhemispheric balance

Stroke typically disrupts the homeostasis of interhemispheric inhibition, leading to suppression of the affected side (36). Crucially, our ANCOVA analysis revealed no significant interaction between the lesioned hemisphere and the treatment outcomes. This indicates that whether the infarction occurred in the left or right hemisphere, the rTMS-integrated therapy consistently facilitated clinical gains. By enhancing the output of the bilateral motor network (as evidenced by increased beta values in fNIRS), rTMS may have counteracted the maladaptive inhibition from the unaffected hemisphere, allowing for more symmetrical motor output, a hypothesis supported by the significant improvement in the gait symmetry index observed in our biomechanical analysis.

### Limitations and future directions

Several limitations warrant consideration. First, while we observed significant improvements at the 6-month follow-up, the long-term persistence of these effects on activities of daily living (ADL) beyond 1 year remains to be verified. Second, fNIRS provides surface cortical data; deeper subcortical changes (e.g., in the basal ganglia) could not be directly observed. Future studies should incorporate multi-modal imaging (e.g., fMRI combined with fNIRS) to map the complete locomotor network. Additionally, exploring stratified rTMS protocols based on individual lesion locations could further optimize precision rehabilitation.

## Conclusion

In conclusion, the integration of rTMS with cognitive-motor dual-task training offers a superior rehabilitation strategy for a specific subgroup of chronic stroke survivors with preserved independent walking ability (FIM ≥ 5), significantly enhancing gait speed, step length, and dual-task capacity. This clinical efficacy is mechanistically supported by the recruitment of the SMA, which acts in concert with improved gait velocity to support complex motor planning. While these findings advocate for the potential inclusion of neuromodulation in standard gait rehabilitation, the results should be interpreted with appropriate caution. Future large-scale, multicenter trials are necessary to confirm these neurophysiological mechanisms and validate the long-term clinical utility of this combined intervention model.

## Data Availability

The original contributions presented in the study are included in the article/supplementary material, further inquiries can be directed to the corresponding author.

## Glossary

ADL: Activities of daily living
ADT: Adaptability test
BDNF: Brain-derived neurotrophic factor
CERT: Consensus on Exercise Reporting Template
CES: Composite equilibrium score
CMI: Cognitive-motor interference
CRP: C-reactive protein
CZ: Central zero
DCL: Directional control
DTUGT: Dual-Task Timed Up and Go Test
EEG: Electroencephalogram
ES: Equilibrium score
FES-I: Falls Efficacy Scale International
FIM: Functional independence measure
FMA: Fugl-Meyer Assessment
fNIRS: Functional near-infrared spectroscopy
HbO: Oxyhemoglobin
ΔHbO: Oxyhemoglobin concentration
LOT: Stability limit test
MMSE: Mini-Mental State Examination
MVL: Movement velocity
MXE: Maximum excursion
10MWT: 10-Meter Walking Test
PMC: Pre-motor cortex
ROI: Regions of interest
ROM: Range of motion
SMA: Supplementary motor area
SMC: Sensorimotor cortex
SOT: Sensory integration test
TMS: Transcranial magnetic stimulation
rTMS - Repetitive transcranial magnetic stimulation
TUGT: Timed Up and Go Test
VFT: Verbal Fluency Test
